# The effect of hawthorn vinegar, black mulberry syrup, and green tea on oral mucositis and oral health-related quality of life in individuals with diabetes: a single-blind, randomized controlled trial

**DOI:** 10.3389/fmed.2026.1841943

**Published:** 2026-08-20

**Authors:** Hatice Demiraǧ, Zahide Akeren

**Affiliations:** 1 Department of Medical Services and Techniques, Kelkit Sema Doǧan Vocational School of Health Services, Gümüşhane University, Gümüşhane, Türkiye; 2 Nursing Department, Health Sciences Faculty, Bayburt University, Bayburt, Türkiye

**Keywords:** black mulberry syrup, green tea, hawthorn vinegar, mucositis, quality of life, Type 2 Diabetes, Glycated Hemoglobin (HbA1c)

## Abstract

**Objective:**

This study was designed as a single-blind randomized controlled trial to investigate the effects of hawthorn vinegar, black mulberry syrup, and green tea on oral mucositis and quality of life related to oral health in individuals with diabetes.

**Methods:**

Total of 120 participants were divided into four groups (hawthorn vinegar, black mulberry syrup, green tea, control; *n* = 30 each) and the study was completed. All participants completed the “Patient Information Form,” “Oral Assessment Guide (OAG/ADR),” and “Oral Health-Related Quality of Life Scale (OHQoL-UK)” before the study, on the 7th day, and after the study. In the experimental groups (hawthorn vinegar group, black mulberry syrup group, green tea group). In addition to routine oral mucositis treatment, patients with DM gargled for 1 min with diluted hawthorn vinegar [1 tablespoon (10 cc) mixed with 40 cc of water], pure black mulberry syrup, or green tea solution three times a day for 14 days, 15–20 min before meals.

**Results:**

Patients in the hawthorn vinegar group showed a decrease in oral mucositis and an increase in oral health-related quality of life at the interim measurement (day 7) and final test (day 14) measurements (*p* < 0.05). A negative correlation was found between the severity of oral mucositis and quality of life (*p* < 0.05).

**Conclusion:**

Although there was no difference between hawthorn vinegar and black mulberry syrup, they were found to be more effective than green tea in reducing oral mucositis and improving oral health-related quality of life, and all three complementary treatments were found to be effective.

**Clinical Trial Registration:**

identifier [NCT06481254]. https://register.clinicaltrials.gov/prs/beta/studies/S000E5O000000043/recordSummary.

## 1 Introduction

Diabetes Mellitus (DM) is a chronic metabolic disease that causes serious damage to the heart, blood vessels, eyes, kidneys, and nerves. Both the number of cases and the prevalence of diabetes have increased rapidly over the past 30 years (1). According to a report by the International Diabetes Federation (IDF) (2021), approximately 537 million people worldwide (expected to reach 700 million by 2045) have diabetes, and 6.5 million people died from diabetes in 2021. In Turkey, it is estimated that approximately 9 million people (expected to reach approximately 13.5 million by 2045) have been diagnosed with diabetes and approximately 80,000 people have died from diabetes (2).

Long-term hyperglycemia in DM leads to microvascular (nephropathy, retinopathy, neuropathy) and macrovascular (hypertension, ischemic heart disease) complications. In addition, poorly controlled diabetes can trigger complications related to immunological and metabolic changes in the oral mucosa (3). Oral findings such as xerostomia, fungal infections, periodontal disease, delayed wound healing, and Burning Mouth Syndrome may be observed in individuals with diabetes; these conditions may predispose to the development of oral mucositis (4).

The development of oral mucositis in diabetes mellitus is a direct consequence of the damage caused to the mucosal tissue by pathophysiological mechanisms. The reduction in Epidermal Growth Factor (EGF) levels in saliva, associated with hyperglycaemia, inhibits re epithelialization in mucosal injuries, thereby halting tissue healing. Furthermore, the inability of macrophages to transition to the M2 phase and their suppression in the pro-inflammatory M1 phenotype leads to continuous cytokine release and, consequently, increased tissue damage. This process, combined with a weakened immune system, causes simple mucosal injuries to develop into chronic, non-healing ulcers. Furthermore, the acidic environment caused by high glucose levels in saliva and the microbial dysbiosis it triggers (such as Candida proliferation) lower mucosal resistance, thereby exacerbating the inflammatory process (5).

Many pharmacological and non-pharmacological agents are used in the prevention/treatment of oral mucositis (6). According to the classification of the National Center for Complementary and Integrative Health (NCCAM), complementary therapy (CT) methods are divided into three groups: nutritional, psychological and physiological, and other complementary approaches (7). However, the literature mentions cryotherapy (oral cooling), honey, propolis, vitamin E, selenium, aloe vera, black mulberry (6), apple cider vinegar, rose water (8), and green tea (9) have been reported to be effective in the treatment of oral mucositis. Again, in the literature, black mulberry syrup (10) in patients with Chronic Obstructive Pulmonary Disease (COPD), a mixture of apple cider vinegar and rose water in cancer patients (8), and green tea (9) have been shown to be effective in treating oral mucositis.

In the literature, there are certain natural products known to improve the pathophysiological process of oral mucositis based on their biological properties (6, 8–10). Hawthorn vinegar, black mulberry syrup and green tea, in particular, are known not only for their anti-glycaemic effects but also for their anti-inflammatory and antioxidant effects on oral mucositis (11–13). It is thought that the polyphenolic content of the hawthorn plant, combined with the antimicrobial effect of the acetic acid in hawthorn vinegar, supports impaired microcirculation in individuals with diabetes and accelerates mucosal healing by increasing tissue perfusion (11). Furthermore, blackberries, which are rich in anthocyanins, suppress the inflammatory response thanks to their ability to neutralize free radicals and inhibit the nuclear factor kappa B (NF-κB) signaling pathway. By combining its antifungal effect against fungal infections commonly seen in diabetic patients with its ability to stimulate epithelialization-which supports tissue repair-black mulberry treats oral mucositis by suppressing the inflammatory response (12). Epigallocatechin-3 -gallate (EGCG) is known to reduce cellular damage by neutralizing reactive oxygen species (ROS), and its polyphenols are known to modulate the excessive activity of matrix metalloproteinases (MMPs)—which cause delayed wound healing in diabetic patients—and to protect the mucosal barrier (13).

The systemic nature of type 2 diabetes critically delays wound healing in the oral mucosa due to oxidative stress caused by hyperglycemia, epidermal growth factor (EGF) deficiency in saliva, and microvascular damage. In this diabetic context, hawthorn vinegar, with its polyphenolic content, offers a specific biological mechanism that supports mucosal repair by improving microcirculation and tissue perfusion impaired by diabetes. Black mulberry syrup suppresses the chronic inflammatory response in diabetic individuals via the NF-κB signaling pathway, while creating an antifungal barrier against opportunistic fungal infections triggered by high glucose levels, thus targeting diabetes-specific complications. Green tea, through its EGCG component, helps maintain tissue integrity by modulating the overactivity of matrix metalloproteinases (MMPs), which accelerate collagen degradation in diabetic wounds. These three interventions regulate impaired tissue regeneration and immune response at the cellular level in diabetic individuals, providing therapeutic efficacy that focuses directly on the pathophysiology of type 2 diabetes, unlike general mucositis protocols. Thus, by addressing the biomechanical deficiencies caused by diabetes, a significant improvement in oral health-related quality of life (OHQoL-UK) is achieved (6–13).

Oral mucositis is one of the oral disorders that can cause oral dysfunction, dysphagia, and a decrease in quality of life related to oral health in patients (14, 15). Oral and dental health affects a person's physiological, psychological, and social functioning, thereby impacting their quality of life. However, improving oral hygiene is part of nursing care (16). Awareness of oral complications arising in patients with DM is necessary to maintain a good quality of life (14). In the reviewed literature, only one experimental study was found in cancer patients evaluating quality of life related to oral mucositis (8). No studies were found on the treatment of oral mucositis in patients with DM, either internationally or in Turkey. Furthermore, there are no studies comparing hawthorn vinegar, black mulberry syrup, and green tea in different sample groups. In this context, the study examined the effects of hawthorn vinegar, black mulberry syrup, and green tea on oral mucositis and oral health-related quality of life in patients with DM.

## 2 Materials and methods

### 2.1 Patient and public involvement

“Patient and Public Involvement” was not included in the study design or in the determination of the research questions and participants. The intervention protocols applied in the study were developed in accordance with clinical guidelines and current literature to ensure the study's safety and standardization. The researcher not responsible for data collection maintained constant communication with participants during the implementation phase to monitor the tolerability and acceptability of the daily use of hawthorn vinegar, black mulberry syrup, and green tea gargle solutions. Participants were specifically monitored throughout the intervention for potential side effects such as oral mucosal irritation, taste intolerance, increased pain, or worsening of existing lesions.

### 2.2 Researcher's objective and type

The study was conducted as a single-blind randomized controlled trial to investigate the effect of hawthorn vinegar, black mulberry syrup, and green tea on oral mucositis and oral health-related quality of life in patients with DM.

### 2.3 Research questions

Question 1: Do hawthorn vinegar, black mulberry syrup, and green tea have an effect on the quality of life related to oral mucositis and oral health in patients with DM?

Question 2: Is there a significant relationship between the severity of oral mucositis (measured by ADR) and the oral health-related quality of life (measured by OHQoL-UK) in individuals with diabetes during the intervention process?

### 2.4 Location and time of the study

Research data was collected from 120 patients with type 2 diabetes who visited the Internal Medicine outpatient clinic of a secondary-level state hospital in a city located in the northeastern region of Türkiye between February 6, 2024, and February 20, 2024, and were admitted to the Internal Medicine Clinic.

### 2.5 Study population and sample

The population of the study consisted of DM patients over the age of 18 who applied to the internal medicine outpatient clinic of a state hospital. Since there were no direct preliminary studies or meta-analyses using these three specific natural interventions simultaneously on oral mucositis in diabetic patients, a “moderate” effect size (effect size of 0.25) was chosen based on Cohen's (17) *F*-tests (ANOVA) criteria to ensure the study's ability to detect clinically significant differences. The sample size was calculated as a total of 128 individuals for 4 groups, considering α = 0.05 error level, 0.25 effect size, and 80% test power with the G^*^Power 3.1.9.7 program. Taking into account possible losses, the study was conducted with 35 participants in each group, totaling 140 participants.

**Figure 1:**
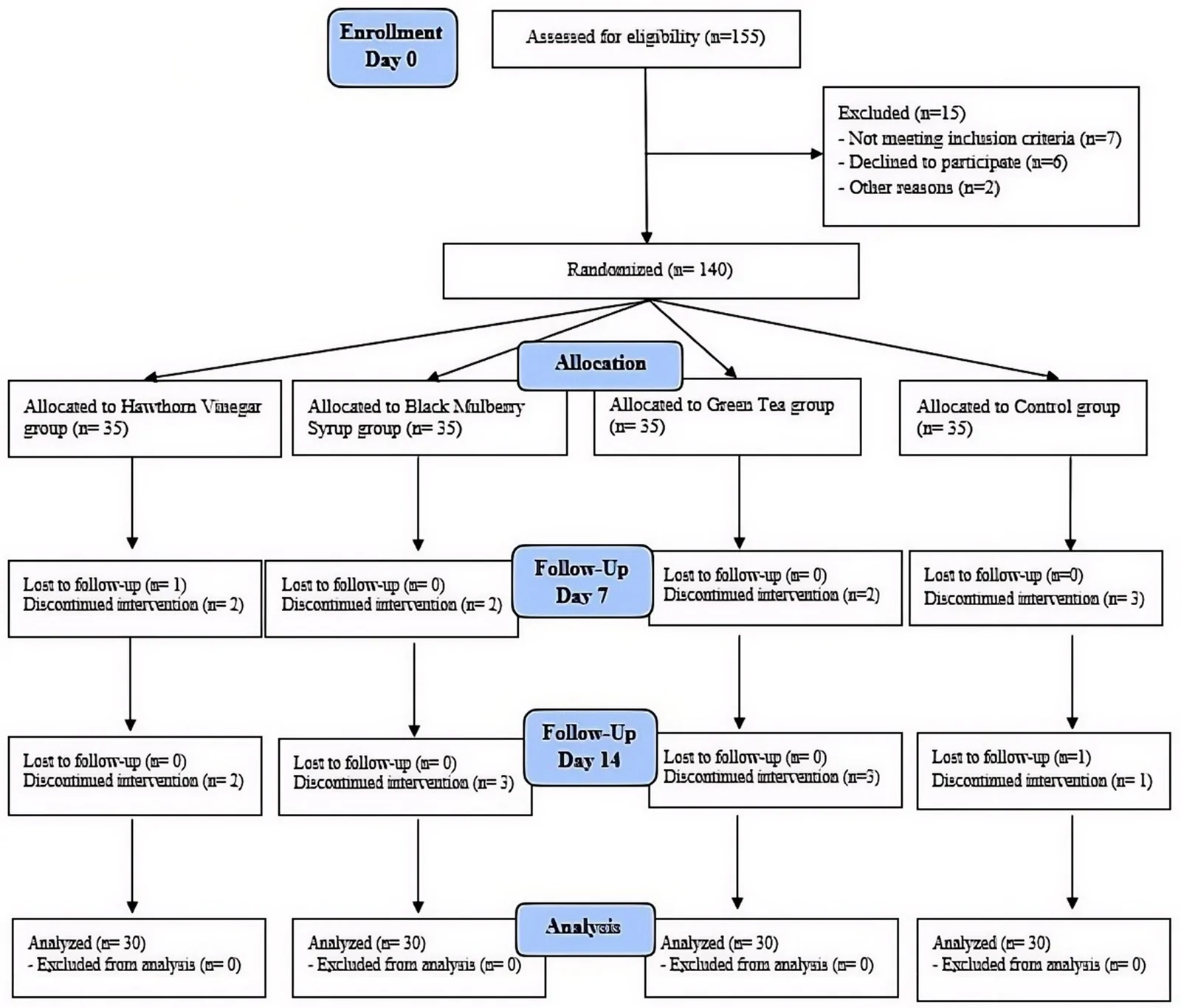
The study CONSORT flow diagram.

As part of the study, 155 patients were initially assessed for eligibility; of these, 15 were excluded (7 for failing to meet inclusion criteria, 6 for refusing to participate, and 2 for other reasons), leaving 140 patients who were randomized into groups. A total of 20 patients were excluded from the study: 9 participants who did not receive the intervention in full during the first 7 days of the follow-up period, 9 participants who discontinued the intervention on day 14, and 2 participants who could not be contacted during follow-up. These exclusions were necessary to maintain the strict methodological integrity of the trial. Consequently, the study was completed with 120 patients (*n* = 30 in each group) who fully completed the study protocol and successfully completed follow-up, and the final analyses were conducted on this sample according to the “per-protocol” principle (Figure 1).

To ensure methodological rigor and minimize selection bias, participants were assigned to one of the four groups (Hawthorn Vinegar, Black Mulberry Syrup, Green Tea, or Control) using a simple randomization procedure. The allocation sequence was generated by an independent statistician not involved in the clinical phase using a computer-generated random number table and was kept in sequentially numbered, opaque, sealed envelopes (SNOSE) to ensure allocation concealment. These envelopes were opened by the investigator only after the participant completed baseline assessments and was formally enrolled. While the distinct sensory properties of the interventions precluded blinding of participants and the primary investigator, the study maintained a single-blind design by ensuring that outcome assessors and data analysts remained strictly blinded to group assignments throughout the study. This separation of roles and the use of an independent assessor were implemented to mitigate potential performance and detection biases. This process ensured that the sampling and randomization stages were distinct and methodologically rigorous. Once participants were selected and the study began, no changes were made to the study protocol. The protocol was followed exactly as originally designed.

#### 2.5.1 Blinding and bias management

Due to the distinct organoleptic properties (taste, smell, and appearance) of hawthorn vinegar, black mulberry syrup, and green tea, absolute participant blinding could not be fully achieved. Therefore, a rigorous single-blind design was implemented focusing on assessor and analyst blinding. The primary outcomes and oral mucositis severity scores were evaluated by an independent clinical assessor who was entirely blinded to the group allocations. Furthermore, the biostatistician performed the statistical analyses using blinded codes (Group A, B, C, and D). To minimize participant-related performance bias, patients were managed individually to prevent cross-communication, and all participants were informed that they were receiving an active natural intervention with potential therapeutic benefits.

#### 2.5.2 Study inclusion criteria

- Being 18 years of age or older,
- Volunteering to participate in the study,
- Being conscious and able to communicate,
- Having been diagnosed with type 2 diabetes mellitus at least 3 months prior,
- Volunteering to use hawthorn vinegar, black mulberry syrup, or green tea as a complementary treatment for oral mucositis,
- Not allergic to hawthorn vinegar, black mulberry syrup, or green tea,
- Type 2 Diabetes patients with Glycated Hemoglobin (HbA1c) ≥ 7% (18),
- Having an oral mucosa score greater than 16 in the oral assessment (19). In this study, the inclusion criterion for participants was defined as a score of 17 or higher according to the Oral Assessment Guide (ADR) scoring developed by Eilers et al., indicating “oral mucosal impairment.” In this classification, a score of 8 is defined as “normal” (healthy), a score of 9–16 as “risk of oral mucosal impairment” (mild-moderate), and a score of 17–24 as “oral mucosal impairment” (severe) (19, 20).

#### 2.5.3 Exclusion criteria for the study

Not meeting the inclusion criteria, having a known psychological disorder, significant systemic comorbidities (e.g., advanced renal failure, active malignancy, or cognitive impairment), HbA1c < 7%, absence of oral mucositis, and hearing loss. However, patients with scores of 16 and below who did not require medical intervention were excluded from the study. To maintain the homogeneity of the study and minimize confounding factors, patients with systemic comorbidities such as advanced renal failure and active malignancy were excluded from the study. One of the primary inclusion criteria was the voluntary agreement to use hawthorn vinegar, black mulberry syrup, or green tea as a complementary treatment for oral mucositis. This criterion functioned as a natural screening process; individuals who had an aversion to the taste or aroma of these specific substances opted not to participate at the recruitment stage.

### 2.6 Data collection tools

Research data were collected using the “Patient Information Form,” “Oral Assessment Guide (ADR),” and “Oral Health-Related Quality of Life Scale (OHQoL-UK).”

#### 2.6.1 Patient information form

Consists of 13 questions regarding patients' sociodemographic characteristics (age, gender, marital status, perceived income level, education level, additional chronic diseases and medication use, smoking use, denture use, tooth brushing, sleep duration, duration of DM, and medication use for DM, etc.) (8–10).

#### 2.6.2 ADR

The Oral Assessment Guide (ADR), developed by Eilers et al. (20), is a tool used to detect oral mucositis and monitor treatment effectiveness. The guide, consisting of eight categories (voice, swallowing, lips, tongue, saliva, mucous membranes, gums, teeth/prostheses), evaluates each category on a scale of 1 (normal) to 3 (severe abnormality). The total score ranges from 8 to 24, with high scores indicating an increased risk or severity of mucositis. In ADR scoring classification, a score of 8 is defined as “normal” (healthy), a score of 9–16 as “risk of oral mucosal impairment” (mild-moderate), and a score of 17–24 as “oral mucosal impairment” (severe) (19, 20). In this study, the Cronbach's alpha values for the OAR were found to be 0.80 in the pre-test, 0.93 in the interim measurement, and 0.97 in the post-test.

#### 2.6.3 OHQoL-UK

OHQoL-UK was developed by McGrath and Bedi (21) and adapted into Turkish by Mumcu et al. (22). The 5-point Likert scale, consisting of a total of 16 items, is designed to determine the degree of impact on teeth, gums, mouth, or dentures and consists of 4 dimensions: “symptoms” (items 1–2), “physical condition” (items 3–7), “psychological condition” (items 8–12), and “social condition” (items 13–16). Each item on the scale is scored as “Very bad effect-1, bad effect-2, no effect-3, good effect-4, Very good effect-5.” The total score of the scale ranges from 16 to 80, and a high score indicates that the individual has a high quality of life related to oral and dental health. The Cronbach's alpha value of the scale was reported as 0.94 (22). In this study, the Cronbach's alpha values of the scale were found to be 0.88 in the pre-test, 0.93 in the interim measurement, and 0.98 in the post-test.

### 2.7 Data collection

The study was designed as a single-blind randomized controlled trial; the researcher collecting the data was kept unaware of the grouping information so that they would not know which group (hawthorn vinegar, black mulberry syrup, green tea, or control) the participants belonged to, thereby reducing the risk of bias.

The 14-day intervention period and the specific assessment points (Day 7 and Day 14) were determined based on the biological healing process of the oral mucosa and evidence-based nursing practices (23). Since the epithelial renewal cycle of the oral cavity is generally completed within 7 to 14 days, these intervals were deemed optimal for capturing both the intermediate (Day 7) and total (Day 14) therapeutic effects of the hawthorn vinegar, black mulberry syrup, and green tea interventions. This timeline is also consistent with existing clinical protocols for treating mucosal inflammation in chronic conditions like diabetes.

All phases of the study, including the administration of interventions (hawthorn vinegar, black mulberry syrup, green tea) and final outcome assessments, were conducted in the same clinical setting. This ensured that all participants were followed under standardized conditions by the same research team throughout the 14-day study period. Safety protocols regarding potential allergic reactions were strictly followed. Although clinical allergy tests were not performed, a detailed medical history was obtained from each participant to identify any known hypersensitivity to the study components. Additionally, participants were instructed to gargle for 1 min and then spit out the solutions instead of swallowing them. Throughout the 14-day period, participants were monitored for any signs of discomfort or allergic symptoms. No adverse events, allergic reactions, or complications were reported or observed during the study period. The procedures applied to each group during the data collection process are described below:

#### 2.7.1 Experimental groups (hawthorn vinegar group, black mulberry syrup group, green tea group)

Before starting the study (pre-test/week 0), all group participants first completed the “Patient Information Form,” the “ADR” to assess the level of oral mucositis in patients, and the “OHQoL-UK.” In the experimental groups (hawthorn vinegar group, black mulberry syrup group, green tea group) In addition to routine oral mucositis [Application of oral hygiene education + the use of a soft-bristle toothbrush + rinsing with 0.9% sodium chloride (saline) 3 times a day] treatment, patients with DM gargled for 1 min with diluted hawthorn vinegar, pure black mulberry syrup, or green tea solution (50 cc) 3 times a day for 14 days, 15–20 min before meals. The “ADR” and “OHQoL-UK” were reapplied on the 7th day (interim measurement) and on the 14th day/end (final test) of the study.

To ensure the reproducibility of the study, all herbal products were prepared according to a standardized protocol. Hawthorn Vinegar was a natural fermentation product with an acidity level of 5%, sourced from a standardized local producer (Gümüşhane, Turkey). For each application, 10 cc (1 tablespoon) of vinegar was diluted with 40 cc of water. Black Mulberry Syrup (Gümüşsu Brand) was a 100% pure extract; participants diluted 10 cc of syrup with 40 cc of water (in a 1:4 ratio) to ensure a consistent concentration. The total sugar content of the Black Mulberry used in the study, at 62.5 g/100 g, consists entirely of fructose and glucose naturally occurring in the fruit, without any added sucrose (refined sugar). The concentrations of the active components, which document the standardization of the product, were determined as follows: total phenolic compounds 0.47%, flavonoid content 0.08%, and antioxidant activity rate 0.77%. Green Tea was prepared by steeping 1 gram (containing approximately 70 mg of epigallocatechin-3-gallate and 130 mg of total catechins) of standardized dried leaves in 50 ml of distilled water at approximately 80 °C for 5 min, and then allowing it to cool to room temperature before use.

#### 2.7.2 Control group

The control group followed the hospital's routine oral mucositis management protocol. Patients in the control group received routine oral mucositis treatment [Application of oral hygiene education + the use of a soft-bristle toothbrush + rinsing with 0.9% sodium chloride (saline) 3 times a day] and no other interventions besides data collection tools. Patients completed the “Patient Information Form,” “ADR,” and “OHQoL-UK” before the study (pre-test/week 0) and again on day 7 (interim measurement) and day 14/end of the study (final test).

To ensure standardization of the study interventions, all procedures were conducted by two researchers holding doctoral degrees in Internal Medicine Nursing. The researcher overseeing the intervention is also a certified specialist in Phytotherapy and Aromatherapy. Although a formal pilot study was not conducted, the intervention protocol was pre-validated through a rigorous review of clinical guidelines and evidence-based nursing practices. The research team held intensive coordination sessions to harmonize patient education, gargling techniques, and assessment methods. Throughout the trial, a structured intervention guide was used to ensure that every participant in the 4 group received the same instructions and monitoring, thus eliminating inter-researcher variability and ensuring methodological consistency. Unlike home-based protocols, all interventions-including the application of hawthorn vinegar, black mulberry syrup, and green tea gargle-were performed in a controlled clinical setting under the direct observation of the research team, with participant adherence strictly documented using a daily structured intervention checklist and clinical follow-up forms.

#### 2.7.3 Standardization of interventions

To ensure scientific reproducibility and rigorous replication, all natural interventions were chemically standardized. The Hawthorn vinegar was a naturally fermented product characterized by a standardized total acidity of 4.5%−5.0% (expressed as acetic acid) and a pH range of 2.8–3.2. The Black mulberry syrup was prepared from 100% pure fruit concentrate standardized to a soluble solids content of 65 °-68 °Brix without any added sucrose or synthetic colorants; its chemical profile documented a total phenolic content of 0.47%, a flavonoid content of 0.08%, and an antioxidant activity rate of 0.77%. The Green tea intervention utilized a standardized dry *Camellia sinensis* leaf extract containing a minimum of 50% total polyphenols [with epigallocatechin-3-gallate (EGCG) comprising at least 30% of the polyphenol fraction], with each dose standardized to contain approximately 70 mg of EGCG and 130 mg of total catechins prepared in a fixed volume of water.

### 2.8 Ethical aspect of the study

Written permission was obtained from the Scientific Research and Publication Ethics Committee of Gümüşhane University and institutional permission was obtained from the X Provincial Health Directorate in order to conduct the research. In addition, written consent was obtained from the patients participating in the study, and permission was obtained from the author who validated the scales in Turkish. This study was conducted in accordance with the Helsinki Declaration, which sets the highest ethical standards for research involving human participants.

### 2.9 Data evaluation

The “*Statistical Package for Social Sciences (SPSS)”* 27.00 program was used to evaluate *the* data. The normality assumption was verified using the Shapiro-Wilk test (*p* > 0.05), and the shape of the distribution was further confirmed as Skewness and Kurtosis values for all variables remained within the acceptable range of −1.5 to +1.5 (24). Homogeneity of variances was confirmed via Levene's test (*p* > 0.05). In the Mixed-Design ANOVA, Mauchly's Test of Sphericity was performed; in cases where the assumption was violated (*p* < 0.05), Greenhouse-Geisser corrections were applied to ensure the reliability of the F-statistics. These steps confirm that all parametric assumptions were strictly met. Mean, standard deviation, number, and percentage were used for descriptive statistics. For categorical variables, the Chi-square test was used for intergroup comparisons. Additionally, while the baseline gender distribution was homogenous across groups (*p* = 0.680), sex-disaggregated subgroup analyses were not performed due to sample size constraints (*n* = 30 per group) and the fact that the biological mechanisms of the natural interventions do not exhibit known sex-specific variations, in alignment with SAGER guidelines. The data were found to be normally distributed. Mean, standard deviation, number, and percentage were used for descriptive statistics. Among parametric tests, the Chi-square test was used for intergroup comparisons. The primary outcome of this study was the severity of oral mucositis, as measured by the Oral Assessment Guide (OAG) scores; the secondary outcome was quality of life, as assessed by the Oral Health-Related Quality of Life Scale (OHQoL-UK). Two types of analytical metrics were used to evaluate the effectiveness of the interventions:

1. Absolute values obtained at three different time points (Day 0-7-14).
2. The amount of change from baseline (mean differences between Day 0 and subsequent measurements). Data are presented as mean ± standard deviation (SD) for all groups. Repeated Measures ANOVA and Mixed ANOVA was used to determine the differences between groups and the change over time, thus analyzing both the effect of time and the total effect of the intervention on the groups. Pearson correlation analysis was used to analyze the relationship between the “ADR” and “OHQoL-UK” means. To evaluate the effectiveness of the interventions, analyses were conducted on a per-protocol basis, including only participants who completed the entire 14-day follow-up. Participants who were not followed up or whose latest data was unavailable were not included in the analysis because data completion was not performed. The final analysis was completed with 120 participants (*n* = 30 per group). The results were calculated at a 95% confidence level and a significance level of 0.05.

## 3 Findings

As part of the study, 155 patients were initially assessed for eligibility; of these, 15 were excluded (7 for failing to meet inclusion criteria, 6 for refusing to participate, and 2 for other reasons), leaving 140 patients who were randomized into groups. A total of 20 patients (14.2%) were excluded from the study: 9 participants who did not receive the intervention in full during the first 7 days of the follow-up period, 9 participants who discontinued the intervention on day 14, and 2 participants who could not be contacted during follow-up. These exclusions were necessary to maintain the strict methodological integrity of the trial. Consequently, the study was completed with 120 patients (*n* = 30 in each group) who fully completed the study protocol and successfully completed follow-up, and the final analyses were conducted on this sample according to the “per-protocol” principle (Figure 1).

Regarding intervention adherence, all procedures were strictly monitored through direct observation by the research team in the clinical setting to ensure standardization. Adherence was documented using daily structured checklists, which verified that each of the 120 participants included in the final analysis (*n* = 30 per group) strictly followed the prescribed dosage, frequency (3 times daily), duration (1-min mouthwash), and timing as outlined in the study protocol. This rigorous monitoring ensured that only participants with 100% compliance were included in the final “per-protocol” analysis. There were no significant differences between the groups in terms of baseline demographic or clinical characteristics (*p* > 0.05), ensuring the comparability of the study arms (Figure 1).

Table 1 shows that there was no difference in the distribution of sociodemographic and disease information between the hawthorn vinegar, black mulberry syrup, green tea, and control groups (*p* > 0.05; Table 1). In addition, the effect size values presented for the relationship between socio-demographic characteristics and disease characteristics within the scope of the study are also shown in Table 1. Baseline demographic and clinical characteristics are presented for the final analyzed population (*N* = 120) based on a per-protocol analysis, after excluding dropouts (*n* = 20) during the follow-up period.

**Table 1:** Sociodemographic and disease characteristics of individuals with diabetes (*N* = 120).

Characteristics	Hawthorn vinegar group (*n* = 30)	Black mulberry syrup group (*n* = 30)	Green tea group (*n* = 30)	Control group (*n* = 30)	F^*^	*p*	*η^2^*
	X ±SD Min-Max	X ±SD Min-Max	X ±SD Min-Max	X ±SD Min-Max			
**Age (years)**	52.63 ± 16.97 (23–85)	54.70 ± 12.33 (35–83)	54.63 ± 14.03 (29–84)	56.90 ± 11.75 (35–80)	0.470	0.202	0.01
	*n* (%)	*n* (%)	*n* (%)	*n* (%)	X^2, **^		Cramer's V
Gender							
Female	14 (46.7)	12 (40.0)	10 (33.3)	14 (46.7)	1,509	0.680	0.11
Male	16 (53.3)	18 (60.0)	20 (66.7)	16 (53.3)			
Marital status							
Married	11 (36.7)	11 (36.7)	12 (40.0)	11 (36.7)	0.107	0.991	0.03
Single (Divorced/Widowed)	19 (63.3)	19 (63.3)	18 (60.0)	19 (63.3)			
Income perception level							
Income is less than expenses	10 (33.3)	12 (40.0)	11 (36.7)	11 (36.7)	0.402	0.999	0.04
Revenue equals expenses	12 (40.0)	11 (36.7)	12 (40.0)	11 (36.7)			
Income exceeds expenses	8 (26.7)	7 (23.3)	7 (23.3)	8 (26.7)			
Education level							
Secondary education and below	7 (23.3)	10 (33.3)	13 (43.3)	12 (40.0)	5,309	0.505	0.14
High school	11 (36.7)	11 (36.7)	12 (40.0)	11 (36.7)			
University and above	12 (40.0)	9 (30.0)	5 (16.7)	7 (23.3)			
Additional chronic disease status							
Yes	19 (63.3)	16 (53.3)	16 (53.3)	17 (56.7)	0.814	0.846	0.08
No	11 (36.7)	14 (46.7)	14 (46.7)	13 (43.3)			
Use of medication for additional chronic disease							
Yes	12 (40.0)	11 (36.7)	9 (30.0)	7 (23.3)	2.241	0.524	0.13
No	18 (60.0)	19 (63.3)	21 (70.0)	23 (76.7)			
Tobacco use							
Yes	15 (50.0)	15 (50.0)	13 (43.3)	16 (53.3)	0.634	0.889	0.07
No	15 (50.0)	15 (50.0)	17 (56.7)	14 (46.7)			
Use of dentures							
Yes	13 (43.3)	14 (46.7)	11 (36.7)	14 (46.7)	0.814	0.846	0.08
No	17 (56.7)	16 (53.3)	19 (63.3)	16 (53.3)			
Daily tooth brushing							
Yes	8 (26.7)	4 (13.3)	4 (13.3)	5 (16.7)	2.482	0.479	0.14
No	22 (73.3)	26 (86.7)	26 (86.7)	25 (83.3)			
Daily sleep hours							
Less than 4 h	5 (16.7)	5 (16.7)	5 (16.7)	6 (20.0)	0.177	1,000	0.02
4–9 h	22 (73.3)	22 (73.3)	22 (73.3)	21 (70.0)			
Over 9 h	3 (10.0)	3 (10.0)	3 (10.0)	3 (10.0)			
DM duration (years)							
Less than 5 years	5 (16.7)	4 (13.3)	5 (16.7)	4 (13.3)	2.466	0.872	0.10
5–10 years	13 (43.3)	18 (60.0)	17 (56.7)	15 (50.0)			
Over 10 years	12 (40.0)	8 (26.7)	8 (26.7)	11 (36.7)			
Use of medication for DM							
OAD medication	13 (43.3)	13 (43.3)	13 (43.3)	13 (43.3)	0.235	1,000	0.03
OAD medication + insulin therapy	9 (30.0)	9 (30.0)	8 (26.7)	8 (26.7)			
Insulin therapy	8 (26.7)	8 (26.7)	9 (30.0)	9 (30.0)			

^*^One-Way ANOVA Test; ^**^Chi-square Test; DM, Diabetes Mellitus; OAD, Oral Antidiabetic; X, Mean; SD, Standard Deviation; ηp2, Partial Eta Squared.

When examining the results of the One-way ANOVA test conducted between groups in Table 2; while there was no significant difference between groups in the pre-test measurements of the ADR's “voice,” “swallowing,” “lips,” “saliva,” and “gums” category mean scores (*p* > 0.05); in the interim measurement, it was determined that the individuals in the hawthorn vinegar and black mulberry syrup group had significantly lower scores than the individuals in the green tea and control groups (*p* < 0.001). In the final test measurement, the ADR “voice,” “swallowing,” “lips,” “saliva,” and “gums” category mean scores were lower in patients in the hawthorn vinegar, Black Mulberry syrup, and green tea groups compared to the control group; while the mean ADR scores for the “voice” category in the hawthorn vinegar group were lower than those in the green tea group (*p* < 0.001; Table 2).

**Table 2:** Comparison of the mean scores of the “Total ADR” and its categories within and between the hawthorn vinegar, black mulberry syrup, green tea, and control groups (*N* = 120).

Subdimension/ Scal	Measurement		Hawthorn vinegar group^a^ (*n* = 30)	Black mulberry syrup group^b^ (*n* = 30)	Green tea group^c^ (*n* = 30)	Control group^d^ (*n* = 30)	*F*	p^**^	*η^2^*	%CI	Multiple comparison
			X ±SD	X ±SD	X ±SD	X ±SD					
**Voice**	Pre-test^1^		2.43 ± 0.50	2.43 ± 0.50	2.36 ± 0.49	2.43 ± 0.50	0.133	0.940	0.003	0.00–0.01	-
	Intermediate measurement^2^		1.63 ± 0.49	1.46 ± 0.57	2.10 ± 0.60	2.40 ± 0.49	18.545	**< 0.001**	0.32	0.17–0.43	a, b < c, d
	Final test^3^		1.10 ± 0.30	1.10 ± 0.30	1.30 ± 0.46	2.56 ± 0.56	82.411	**< 0.001**	0.68	0.57–0.74	a, b, c < d; a < c
	**%CI**		1.58–1.86	1.52–1.80	1.78–2.06	2.32–2.60					
	**Intra-group analysis** ^*^	***sd***	2	2	2	2					
		***F***	84.667	90.047	60.214	1.701					
		***p***	**< 0.001**	**< 0.001**	**< 0.001**	0.187					
		* $\eta_{p}^{2}$ *	0.59	0.61	0.51	0.02					
	**Significant difference** ^***^		1–2 (***p*** **<** **0.001**) 2–3 (***p*** **<** **0.001**) 1–3 (***p*** **<** **0.001**)	1–2 (***p*** **<** **0.001**) 2–3 (***p*** **<** **0.001**) 1-3 (***p*** **<** **0.001)**	1–2 (***p*** **=** **0.016**) 2–3 (***p*** **<** **0.001**) 1–3 (***p*** **<** **0.001)**	1–2 (*p* = 1.000) 2–3 (*p* = 0.217) 1–3 (*p* = 0.590)					
	**Intergroup analysis** ^****^			***Sd*** **=** 6	*F* = 30.587; ***p*** **<** **0.001**		*$\eta_{p}^{2}$* = 0.44			
**Swallowing**	Pre-test^1^		2.46 ± 0.50	2.46 ± 0.57	2.40 ± 0.49	2.26 ± 0.52	0.967	0.411	0.02	0.00–0.08	-
	Intermediate measurement^2^		1.66 ± 0.47	1.50 ± 0.57	2.10 ± 0.60	2.36 ± 0.55	15.278	**0.001**	0.28	0.14–0.39	a, b < c, d
	Final test^3^		1.05 ± 0.25	1.13 ± 0.34	1.33 ± 0.47	2.46 ± 0.57	69.408	**0.001**	0.64	0.53–0.70	a, b, c < d
	**%CI**		1.59–1.87	1.56–1.83	1.80–2.08	2.22–2.50					
	**Intra-group analysis** ^*^	***sd***	2	2	2	2					
		***F***	75.982	72.860	50.209	1.557					
		***p***	**< 0.001**	**< 0.001**	**< 0.001**	0.215					
		* $\eta_{p}^{2}$ *	0.56	0.55	0.46	0.02					
	**Significant difference** ^***^		1–2 (***p*** **<** **0.001**) 2–3 (***p*** **<** **0.001**) 1–3 (***p*** **<** **0.001**)	1–2 (***p*** **<** **0.001**) 2–3 (***p*** **<** **0.001**) 1–3 (***p*** ** < 0.001)**	1–2 (***p*** **=** **0.013**) 2–3 (***p*** **<** **0.001**) 1–3 (***p*** **<** **0.001)**	1–2 (*p* = 0.998) 2–3 (*p* = 0.903) 1–3 (*p* = 0.239)					
	**Intergroup analysis** ^****^			***Sd*** **=** **6**	*F* = 28.693; ***p*** **<** **0.001**		*$\eta_{p}^{2}$* = 0.42			
**Lips**	Pre-test^1^		2.40 ± 0.56	2.43 ± 0.50	2.36 ± 0.49	2.26 ± 0.58	0.540	0.656	0.01	0.00–0.05	-
	Intermediate measurement^2^		1.56 ± 0.50	1.43 ± 0.56	2.06 ± 0.63	2.36 ± 0.55	17.524	**0.001**	0.31	0.16–0.41	a, b < c, d
	Final test^3^		1.06 ± 0.25	1.10 ± 0.30	1.33 ± 0.47	2.56 ± 0.56	85.154	**< 0.001**	0.68	0.58–0.74	a, b, c < d
	**%CI**		1.53–1.82	1.50–1.80	1.77–2.07	2.25–2.54					
	**Intra-group analysis** ^*^	***sd***	2	2	2	2					
		***F***	84.954	90.184	55.037	4.510					
		***p***	**< 0.001**	**< 0.001**	**< 0.001**	**< 0.001**					
		* $\eta_{p}^{2}$ *	0.59	0.61	0.48	0.07					
	**Significant difference** ^***^		1–2 (***p*** **<** **0.001**) 2–3 (***p*** **<** **0.001**) 1–3 (***p*** **<** **0.001**)	1–2 (***p*** **<** **0.001**) 2–3 (***p*** **=** **0.002**) 1–3 (***p*** **<** **0.001)**	1–2 (***p*** **=** **0.008**) 2–3 (***p*** **<** **0.001**) 1–3 (***p*** **<** **0.001)**	1–2 (*p* = 0.923) 2–3 (*p* = 0.107) 1–3 **(*****p*** **=** **0.012)**					
	**Intergroup analysis** ^****^			**Sd** **=** 5.94	*F* = 35.239; ***p*** **<** **0.001**		*$\eta_{p}^{2}$* = 0.47			
**Language**	Pre-test^1^		2.50 ± 0.50	2.43 ± 0.50	2.43 ± 0.50	2.30 ± 0.59	0.753	0.523	0.01	0.00–0.06	-
	Intermediate measurement^2^		1.56 ± 0.50	1.53 ± 0.50	2.23 ± 0.56	2.30 ± 0.53	18.431	**0.001**	0.32	0.17–0.42	a, b < c, d
	Final test^3^		1.00 ± 0.00	1.10 ± 0.30	1.36 ± 0.49	2.53 ± 0.57	90.671	**0.001**	0.70	0.60–0.75	a < c, d; b, c < d
	**%CI**		1.55–1.87	1.55–1.82	1.87–2.14	2.24–2.51					
	**Intra-group analysis** ^*^	***sd***	2	2	2	2					
		***F***	109.390	89.445	58.341	3.352					
		***p***	**< 0.001**	**< 0.001**	**< 0.001**	0.074					
		* $\eta_{p}^{2}$ *	0.65	0.60	0.50	0.05					
	**Significant difference** ^***^		1–2 (***p*** **<** **0.001**) 2–3 (***p*** **<** **0.001**) 1–3 (***p*** **<** **0.001**)	1–2 (***p*** **<** **0.001**) 2–3 (***p*** **<** **0.001**) 1–3 (***p*** **<** **0.001)**	1–2 (*p* = 0.094) 2–3 (***p*** **<** **0.001**) 1–3 (***p*** **<** **0.001)**	1–2 (*p* = 1.000) 2–3 (*p* = 0.057) 1–3 (*p* = 0.079)					
	**Intergroup analysis** ^****^			***Sd*** **=** 6	*F* = 36.926; ***p*** **<** **0.001**		*$\eta_{p}^{2}$* = 0.48			
			**X** ±**SD**	**X** ±**SD**	**X** ±**SD**	**X** ±**SD**					
**Saliva**	Pre-test^1^		2.36 ± 0.55	2.53 ± 0.50	2.33 ± 0.47	2.33 ± 0.50	1.777	0.155	0.04	0.00–0.11	-
	Intermediate Measurement^2^		1.46 ± 0.50	1.50 ± 0.50	2.06 ± 0.69	2.30 ± 0.53	16.175	**0.001**	0.29	0.15–0.40	a, b < c, d
	Final Test^3^		1.10 ± 0.30	1.06 ± 0.25	1.30 ± 0.46	2.46 ± 0.57	75.368	**0.001**	0.66	0.55–0.72	a, b, c < d
	**%CI**		1.50–1.78	1.55–1.84	1.75–2.04	2.19–2.47					
	**Intra-group analysis** ^*^	***sd***	2	2	2	2					
		***F***	81.213	108.380	52.707	2.640					
		***p***	**< 0.001**	**< 0.001**	**< 0.001**	0.076					
		* $\eta_{p}^{2}$ *	0.58	0.65	0.47	044					
	**Significant difference** ^***^		1–2 (***p*** **<** **0.001**) 2–3 (***p*** **<** **0.001**) 1–3 (***p*** **<** **0.001**)	1–2 (***p*** **<** **0.001**) 2–3 (***p*** **<** **0.001**) 1–3 (***p*** **<** **0.001)**	1–2 (***p*** **=** **0.013**) 2–3 (***p*** **<** **0.001**) 1–3 (***p*** **<** **0.001)**	1–2 (*p* = 1.000) 2–3 (*p* = 0.253) 1–3 (*p* = 0.080)					
	**Intergroup analysis** ^****^			***Sd*** **=** 6	*F* = 35.410; ***p*** **<** **0.001**		*$\eta_{p}^{2}$* = 0.47			
**Oral mucosa**	Pre-test^1^		2.46 ± 0.50	2.60 ± 0.49	2.46 ± 0.50	2.20 ± 0.25	3.167	**0.027**	0.07	0.00–0.16	d < b
	Intermediate measurement^2^		1.40 ± 0.49	1.46 ± 0.50	2.26 ± 0.63	2.36 ± 0.55	25.732	**0.001**	0.40	0.25–0.49	a, b < c, d
	Final test^3^		1.03 ± 0.18	1.00 ± 0.00	1.43 ± 0.50	2.60 ± 0.56	111.222	**0.001**	0.74	0.65–0.79	a, b < c, d; c < d
	**%CI**		1.49–1.77	1.54–1.83	1.93–2.19	2.24–2.53					
	**Intra-group analysis** ^*^	***sd***	2	2	2	2					
		***F***	127.485	155.112	66.640	9.021					
		***p***	**< 0.001**	**< 0.001**	**< 0.001**	**< 0.001**					
		* $\eta_{p}^{2}$ *	0.68	0.73	0.53	0.13					
	**Significant difference** ^***^		1–2 (***p*** **<** **0.001**) 2–3 (***p*** **<** **0.001**) 1–3 (***p*** **<** **0.001**)	1–2 (***p*** **<** **0.001**) 2–3 (***p*** **<** **0.001**) 1–3 (***p*** **<** **0.001)**	1–2 (*p* = 0.086) 2–3 (***p*** **<** **0.001**) 1–3 (***p*** **<** **0.001)**	1–2 (*p* = 0.201) 2–3 **(*****p*** **=** **0.040)** 1–3 (***p*** **<** **0.001)**					
	**Intergroup analysis** ^****^			***Sd*** **=** 5.98	*F* = 43.117; ***p*** **<** **0.001**	*$\eta_{p}^{2}$* = 0.59				
			**X** ±**SD**	**X** ±**SD**	**X** ±**SD**	**X** ±**SD**					
**Gums**	Pre-test^1^		2.40 ± 0.62	2.53 ± 0.50	2.33 ± 0.47	2.23 ± 0.50	1.685	0.174	0.04	0.00–0.11	-
	Intermediate measurement^2^		1.33 ± 0.47	1.50 ± 0.50	2.06 ± 0.69	2.30 ± 0.53	20.078	**0.001**	0.34	0.19–0.44	a, b < c, d
	Final test^3^		1.06 ± 0.25	1.06 ± 0.25	1.30 ± 0.46	2.46 ± 0.57	80.159	**0.001**	0.67	0.57–0.73	a, b, c < d
	**%CI**		1.45–1.74	1.55–1.84	1.75–2.04	2.18–2.47					
	**Intra-group analysis** ^*^	***sd***	2	2	2	2					
		***F***	93.063	104.029	52.896	2.641					
		***p***	**< 0.001**	**< 0.001**	**< 0.001**	0.076					
		* $\eta_{p}^{2}$ *	0.61	0.64	0.47	0.04					
	**Significant difference** ^***^		1–2 (***p*** **<** **0.001**) 2–3 (***p*** **=** **0.016**) 1–3 (***p*** **<** **0.001**)	1–2 (**p < 0.001**) 2–3 (***p*** **<** **0.001**) 1–3 (***p*** **<** **0.001)**	1–2 (***p*** **=** **0.016**) 2–3 (***p*** **<** **0.001**) 1–3 (***p*** **<** **0.001)**	1–2 (*p* = 1.00) 2–3 (*p* = 0.237) 1–3 (*p* = 0.085)					
	**Intergroup analysis** ^****^			***Sd*** **=** 6	*F* = 37.446; ***p*** **<** **0.001**	*$\eta_{p}^{2}$* = 0.49				
**Teeth/prosthesis**	Pre-test^1^		2.30 ± 0.53	2.56 ± 0.50	2.33 ± 0.50	2.30 ± 0.59	1.665	0.178	0.04	0.00–0.11	-
	Mid-term measurement^2^		1.33 ± 0.47	1.43 ± 0.50	2.10 ± 0.71	2.40 ± 0.62	23.271	**0.001**	0.37	0.22–0.47	a, b < c, d
	Final test^3^		1.06 ± 0.25	1.06 ± 0.25	1.40 ± 0.56	2.53 ± 0.57	75.204	**0.001**	0.66	0.55–0.72	a, b < c, d; c < d
	**%CI**		1.41–1.72	1.53–1.84	1.79–2.09	2.25–2.09					
	**Intra-group analysis** ^*^	***sd***	2	2	2	2					
		***F***	81.755	118.406	45.573	2.621					
		***p***	**< 0.001**	**< 0.001**	**< 0.001**	0.077					
		* $\eta_{p}^{2}$ *	0.58	0.67	0.44	0.04					
	**Significant difference** ^***^		1–2 (***p*** **<** **0.001**) 2–3 (***p*** **=** **0.020**) 1–3 (***p*** **<** **0.001**)	1–2 (***p*** **<** **0.001**) 2–3 (***p*** **<** **0.001**) 1–3 (***p*** **<** **0.001)**	1–2 (***p*** **=** **0.052**) 2–3 (***p*** **<** **0.001**) 1–3 (***p*** **<** **0.001)**	1–2 (*p* = 0.909) 2–3 (*p* = 0.513) 1–3 (*p* = 0.071)					
	**Intergroup analysis** ^****^			***Sd*** **=** 6	*F* = 35.986; ***p*** **<** **0.001**	*$\eta_{p}^{2}$* = 0.48				
			**X** ±**SD**	**X** ±**SD**	**X** ±**SD**	**X** ±**SD**					
**Total ADR** ^***^	Pre-test^1^		19.33 ± 2.96	20.0 ± 2.66	19.03 ± 2.53	18.23 ± 2.67	2.184	0.094	0.05	0.00–0.13	-
	Intermediate measurement ^2^		11.96 ± 2.91	11.83 ± 3.15	17.00 ± 4.05	18.80 ± 3.11	33.734	**0.001**	0.46	0.32–0.55	a, b < c, d
	Final test^3^		8.50 ± 1.61	8.63 ± 1.65	10.76 ± 2.84	20.20 ± 3.69	136.173	**0.001**	0.77	0.70–0.82	a, b < c, d; c < d
	**%CI**		12.47–14.06	12.69–14.28	14.80–16.39	18.28–19.87					
	**Intra-group analysis** ^*^	***sd***	2	2	2	2					
		***F***	161.221	181.416	97.345	5.368					
		***p***	**< 0.001**	**< 0.001**	**< 0.001**	0.006					
		* $\eta_{p}^{2}$ *	0.73	0.75	0.62	0.08					
	**Significant difference** ^***^		1–2 (***p*** **<** **0.001**) 2–3 (***p*** **=** **0.020**) 1–3 (***p*** **<** **0.001**)	1–2 (***p*** **<** **0.001**) 2–3 (***p*** **<** **0.001**) 1–3 (***p*** **<** **0.001)**	1–2 (***p*** **=** **0.002**) 2–3 (***p*** **<** **0.001**) 1–3 (***p*** **<** **0.001)**	1–2 (*p* = 1.000) 2–3 (*p* = 0.062) 1–3 (*p* = 0.005)					
	**Intergroup analysis** ^****^			**Sd** **=** 6	*F* = 59.653; ***p*** **<** **0.001**	*$\eta_{p}^{2}$*= 0.61				

^*^Repeated measures ANOVA test; ^**^One-Way ANOVA test, ^***^Bonferroni test for multiple comparisons in repeated measures ANOVA; ^****^Mixed-design ANOVA test; ADR, Oral Assessment Guide; η2, Eta Squared; ηp2, Partial Eta Squared; CI, Confidence Interval. Bold *p*-values indicate that the *p*-value is statistically significant. a: Hawthorn vinegar group, b: Black mulberry syrup group, c: Green tea group, d: Control group, 1: Pre-test, 2: Intermediate measurement, 3: Final test.

ADR “language” category mean scores showed no significant differences between groups in the pre-test measurement (*p* > 0.05); however, mean scores in the interim measurement were found to be significantly lower in individuals in the hawthorn vinegar and black mulberry syrup groups compared to those in the tea and control groups (*p* < 0.001). The final test mean scores were found to be significantly lower in individuals in the hawthorn vinegar group compared to those in the green tea and control groups, and in patients in the black mulberry and green tea groups compared to those in the control group (*p* < 0.001; Table 2).

Table 2 shows that there was no significant difference between groups in the ADR “teeth/prosthetics” category and “total ADR” pre-test measurements (*p* > 0.05); ADR “oral mucosa” category pre-test mean scores were significantly lower in individuals in the control group compared to individuals in the black mulberry group; ADR “oral mucosa,” “teeth/prosthetics” category, and “total ADR” were significantly lower in the hawthorn vinegar and black mulberry syrup groups compared to the green tea and control groups in the interim and final test measurements; again, in the final test measurements, it was determined that the scores in the green tea group were lower than in the control group (*p* < 0.001; Table 2).

According to the results of repeated measures ANOVA and mix-design ANOVA, which was conducted to determine the changes in the groups over time and the effect of the interventions; a significant decrease was recorded in the total ADR scores and all subcategories over time in all experimental groups consisting of hawthorn vinegar, black mulberry syrup, and green tea (*p* < 0.05). Although a general decreasing trend was observed in the “language” and “oral mucositis” categories, the difference between the pre-test and interim measurement (day 7) was found not to be statistically significant in the green tea group (*p* > 0.05). In the control group, a non-significant increase over time was observed in the total ADR score and in all subcategories except “lips” and “oral mucosa” (*p* > 0.05). However, a significant increase was determined between pre-test and post-test in the “lips” category, and between both pre-test and post-test measurements, and between interim and post-test measurements in the “oral mucositis” category (*p* < 0.05; Table 2).

When examining the results of the one-way ANOVA test conducted between groups; no significant difference was found between groups in the pre-test measurements of the OHQoL-UK “symptom” and “psychological” subdimensions (*p* > 0.05); The pre-test mean scores for the “physical condition” subscale were significantly lower in individuals in the hawthorn vinegar group compared to those in the control group; The pre-test mean scores for the “social condition” subscale were significantly lower in patients in the hawthorn vinegar and black mulberry syrup groups compared to individuals in the control group; The pre-test mean scores for “Total OHQoL-UK” were found to be significantly lower in patients in the hawthorn vinegar group compared to individuals in the control group (*p* < 0.05). OHQoL-UK “symptom,” “physical condition,” “psychological condition,” “social condition” subscale, and “Total OHQoL-UK” interim measurement mean scores were significantly higher in individuals in the hawthorn vinegar and black mulberry syrup groups compared to individuals in the green tea and control groups; The OHQoL-UK “symptoms,” “psychological state” subscale, and “Total OHQoL-UK” interim measurement mean scores were significantly higher in the green tea group than in the control group (*p* < 0.05). The mean scores for the OHQoL-UK “symptoms,” “physical condition,” “psychological condition,” “social condition” subscales, and “Total OHQoL-UK” final test measurements were significantly higher in individuals in the hawthorn vinegar and black mulberry syrup groups compared to those in the green tea and control groups; while in the green tea group, they were significantly higher than in the control group (*p* < 0.05; Table 3).

**Table 3:** Comparison of intra-group and inter-group mean scores for “Total OHQoL-UK” and subdimensions in hawthorn vinegar, black mulberry syrup, green tea, and control groups (*N* = 120).

Subdimension/ Scal	Measurement		Hawthorn vinegar group^a^ (*n* = 30)	Black Mulberry Syrup group^b^ (*n* = 30)	Green tea group^c^ (*n* = 30)	Control group^d^ (*n* = 30)	*F*	p^**^	*η^2^*	%CI	Multiple comparison
			X ±SD	X ±SD	X ±SD	X ±SD					
**Symptom**	Pre-test^1^		3.03 ± 0.96	3.10 ± 0.88	3.16 ± 0.91	3.23 ± 1.13	0.232	0.874	0.006	0.00–0.03	-
	Intermediate measurement^2^		4.73 ± 1.14	4.86 ± 1.00	3.53 ± 1.04	2.80 ± 0.92	27.644	**< 0.001**	0.41	0.27–0.51	c, d < a, b; d < c
	Final test^3^		7.13 ± 1.50	7.33 ± 1.32	5.53 ± 1.04	2.53 ± 0.86	101.271	**< 0.001**	0.72	0.63–0.77	c, d < a, b; d < c
	**%CI**		4.62–5.31	4.75–5.44	3.73–4.42	2.51–3.20					
	**Intra-group analysis** ^*^	***sd***	2	2	2	2					
		***F***	220.791	235.395	94.210	7.563					
		***p***	**< 0.001**	**< 0.001**	**< 0.001**	**< 0.001**					
		* $\eta_{p}^{2}$ *	0.79	0.80	0.62	0.11					
	**Significant difference** ^***^		1–2 (***p*** **<** **0.001**) 2–3 (***p*** **<** **0.001**) 1–3 (***p*** **<** **0.001**)	1–2 (***p*** **<** **0.001**) 2–3 (***p*** **<** **0.001**) 1–3 (***p*** **<** **0.001)**	1–2 (***p*** **=** **0.012**) 2–3 (***p*** **<** **0.001**) 1–3 (***p*** **<** **0.001)**	1–2 (***p*** **=** **0.002**) 2–3 ( = 0.224) 1–3 (***p*** **=** **0.001)**					
	**Intergroup analysis** ^****^		***Sd*** **=** 4.69	*F* = 107.603; ***p*** **<** **0.001**		*$\eta_{p}^{2}$* = 0.73			
**Physical condition**	Pre-test^1^		7.36 ± 1.90	7.56 ± 2.41	8.46 ± 2.40	9.23 ± 3.01	3.664	**0.014**	0.008	0.00–0.17	a < d
	Intermediate measurement ^2^		12.03 ± 2.32	12.46 ± 2.06	9.66 ± 3.03	8.03 ± 2.39	21.099	**< 0.001**	0.35	0.20–0.45	c, d < a, b
	Final test^3^		17.80 ± 3.65	18.80 ± 2.99	15.16 ± 3.15	6.70 ± 1.98	100.096	**< 0.001**	0.72	0.62–0.77	c, d < a, b; d < c
	**%CI**		11.55–13.24	12.09–13.79	10.25–11.94	7.14–8.83					
	**Intra-group analysis** ^*^	***sd***	2	2	2	2					
		***F***	*F* = 218.216	*F* = 253.121	*F* = 118.565	*F* = 12.902					
		***p***	**< 0.001**	**< 0.001**	**< 0.001**	**< 0.001**					
		* $\eta_{p}^{2}$ *	0.79	0.81	0.67	0.18					
	**Significant difference** ^***^		1–2 (***p*** **<** **0.001**) 2–3 (***p*** **<** **0.001**) 1–3 (***p*** **<** **0.001**)	1–2 (***p*** **<** **0.001**) 2–3 (***p*** **<** **0.001**) 1–3 (***p*** **<** **0.001)**	1–2 (*p* = 0.001) 2–3 (***p*** **<** **0.001**) 1–3 (***p*** **<** **0.001**)	1–2 (*p* = 0.001) 2–3 (*p* = 0.001) 1–3 (***p*** **<** **0.001)**					
	**Intergroup analysis** ^****^		***Sd*** **=** 4.65	*F* = 126.841; ***p*** **<** **0.001**		*$\eta_{p}^{2}$* = 0.76			
**Psychological state**	Pre-test^1^		7.70 ± 1.85	8.20 ± 1.74	8.40 ± 2.23	8.96 ± 2.90	1.649	0.182	0.04	0.00–0.11	-
	Intermediate measurement^2^		12.20 ± 2.15	12.83 ± 1.83	9.66 ± 2.77	7.53 ± 2.19	34.729	**< 0.001**	0.47	0.33–0.56	c, d < a, b; d < c
	Final test^3^		18.26 ± 2.86	19.00 ± 2.59	14.86 ± 2.52	6.70 ± 1.93	151.830	**< 0.001**	0.79	0.72–0.83	c, d < a, b; d < c
	**%CI**		11.98–13.46	12.60–14.08	10.23–11.71	6.99–8.47					
	**Intra-group analysis** ^*^	***sd***	2	2	2	2					
		***F***	291.535	304.312	139.330	15.011					
		***p***	**< 0.001**	**< 0.001**	**< 0.001**	**< 0.001**					
		* $\eta_{p}^{2}$ *	0.83	0.84	0.70	0.20					
	**Significant difference** ^***^		1–2 (***p*** **<** **0.001**) 2–3 (***p*** **<** **0.001**) 1–3 (***p*** **<** **0.001**)	1–2 (***p*** **<** **0.001**) 2–3 (***p*** **<** **0.001**) 1–3 (***p*** **<** **0.001)**	1–2 (***p*** **<** **0.001**) 2–3 (***p*** **<** **0.001**) 1–3 (***p*** **<** **0.001**)	1–2 (***p*** **<** **0.001**) 2–3 (*p* = 0.031) 1–3 (***p*** **<** **0.001)**					
	**Intergroup analysis** ^****^		***Sd*** **=** 4.75	*F* = 149.838; ***p*** **<** **0.001**		*$\eta_{p}^{2}$* = 0.79			
**Social status**	Pre-test^1^		6.20 ± 1.34	6.06 ± 1.52	6.56 ± 1.43	7.40 ± 1.84	4.449	**0.005**	0.10	0.01–0.19	a, b < d
	Intermediate measurement^2^		9.96 ± 1.82	9.93 ± 1.74	7.50 ± 1.90	6.26 ± 1.68	31.682	**< 0.001**	0.45	0.30–0.54	c, d < a, b
	Final test^3^		14.73 ± 2.30	15.00 ± 2.27	11.66 ± 1.74	5.46 ± 1.63	145.483	**< 0.001**	0.79	0.71–0.82	c, d < a, b; d < c
	**%CI**		9.72–10.87	9.76–10.90	8.00–9.14	5.80–6.94					
	**Intra-group analysis** ^*^	***sd***	2	2	2	2					
		***F***	364.701	398.039	139.630	21.295					
		***p***	**< 0.001**	**< 0.001**	**< 0.001**	**< 0.001**					
		* $\eta_{p}^{2}$ *	0.86	0.87	0.70	0.27					
	**Significant difference** ^***^		1–2 (***p*** **<** **0.001**) 2–3 (***p*** **<** **0.001**) 1–3 (***p*** **<** **0.001**)	1–2 (***p*** **<** **0.001**) 2–3 (***p*** **<** **0.001**) 1–3 (***p*** **<** **0.001)**	1–2 (***p*** **<** **0.001**) 2–3 (***p*** **<** **0.001**) 1–3 (***p*** **<** **0.001**)	1–2 (***p*** **<** **0.001**) 2–3 (*p* = 0.012) 1–3 (***p*** **<** **0.001)**					
	**Intergroup analysis** ^****^		***Sd*** **=** 5.03	*F* = 175.526; ***p*** **<** **0.001**		*$\eta_{p}^{2}$* = 0.81			
**Total OHQoL-UK** ^***^	Pre-test^1^		24.30 ± 5.51	24.93 ± 5.72	26.60 ± 5.73	28.83 ± 7.76	3.147	**0.028**	0.07	0.00–0.16	a < d
	Intermediate measurement ^2^		38.93 ± 6.73	40.10 ± 5.80	30.36 ± 7.54	24.63 ± 6.00	37.543	**< 0.001**	0.49	0.35–0.58	c, d < a, b; d < c
	Final test^3^		57.93 ± 9.55	60.13 ± 8.58	47.23 ± 7.00	21.40 ± 5.44	155,470	**< 0.001**	0.80	0.73–0.83	c, d < a, b; d < c
	**%CI**		38.21–42.56	39.54–43.89	32.55–36.90	22.78–27.13					
	**Intra-group analysis** ^*^	***sd***	2	2	2	2					
		***F***	321.282	351.995	154.796	16.736					
		***p***	**< 0.001**	**< 0.001**	**< 0.001**	**< 0.001**					
		* $\eta_{p}^{2}$ *	0.84	0.86	0.72	0.22					
	**Significant difference** ^***^		1–2 (***p*** **<** **0.001**) 2–3 (***p*** **<** **0.001**) 1–3 (***p*** **<** **0.001**)	1–2 (***p*** **<** **0.001**) 2–3 (***p*** **<** **0.001**) 1–3 (***p*** **<** **0.001)**	1–2 (***p*** **<** **0.001**) 2–3 (***p*** **<** **0.001**) 1–3 (***p*** **<** **0.001**)	1–2 (***p*** **<** **0.001**) 2–3 (*p* = 0.004) 1–3 (***p*** **<** **0.001)**					
	**Intergroup analysis** ^****^		***Sd*** **=** 4.63	*F* = 174.334; ***p*** **<** **0.001**		*$\eta_{p}^{2}$* = 0.82			

^*^Repeated measures ANOVA test, ^**^One-Way ANOVA test, ^***^Bonferroni test for multiple comparisons in repeated measures ANOVA; ^****^Mixed-design ANOVA test.

ADR, Oral Assessment Guide; η2, Eta Squared; ηp2, Partial Eta Squared; CI, Confidence Interval. Bold *p*-values indicate that the *p*-value is statistically significant. a: Hawthorn vinegar group, b: Black mulberry syrup group, c: Green tea group, d: Control group, 1: Pre-test, 2: Intermediate measurement, 3: Final test.

According to the results of repeated measures ANOVA and mix-design ANOVA, which was conducted to determine the changes in the groups over time and the effect of the interventions; it was determined that the total OHQoL-UK scores and the mean scores of all sub-dimensions increased significantly over time in all experimental groups consisting of hawthorn vinegar, black mulberry syrup, and green tea; while they decreased significantly in the control group (*p* < 0.05). However, the difference between the pre-test and interim (days 0–7) scores of the “physical condition” sub-dimension in the patients in the green tea group was not statistically significant (*p* > 0.05). In the patients in the control group, there was no significant increase in the OHQoL-UK “symptom,” “psychological condition,” “social condition” sub-dimensions and the “total OHQoL-UK” interim and post-test measurements; and in the “physical condition” sub-dimension, there was no significant increase between the pre-test and post-test measurements or between the interim and post-test measurements (*p* > 0.05; Table 3).

According to the Pearson correlation test conducted to determine the relationship between oral assessment and oral health-related quality of life, a moderate negative correlation was found between the mean total ADR and total OHQoL-UK mid-test scores, and a strong negative correlation was found between the mean final test scores (*p* < 0.05). However, no significant relationship was found between the mean pre-test scores of total ADR and total OHQoL-UK (*p* > 0.05; Table 4).

**Table 4:** Relationship between patients' “Total ADR” and “Total OHQoL-UK” (*N* = 120).

Measurement	OHQoL-UK pre-test		OHQoL-UK mid-measurement		OHQoL-UK post-test	
	*r*	*p*	*r*	*p*	*r*	*p*
ADR pre-test	0.075	0.415				
ADR interim measurement			−0.503	**< 0.001** ^*^		
ADR last measurement					−0.754	**< 0.001** ^*^

ADR, Oral Assessment Guide; OHQoL-UK, Oral Health-Related Quality of Life Scale-United Kingdom; r, Pearson Correlation Test; ^*^Significance level p < 0.001. Bold *p*-values indicate that the *p*-value is statistically significant.

Table 5 shows that the time^*^group interaction had a statistically significant effect on the “total ADR” (*F* = 59.653) and “total OHRQoL-UK” (*F* = 174.334) scores (*p* < 0.001). However, the time^*^gender and time^*^group^*^gender interactions were not significant (*p* > 0.05); and it was determined that the gender factor did not play a determining role in the changes in “total ADR” and “total OHRQoL-UK” scores (Table 5).

**Table 5:** Comparison of “Total ADR” and “Total OHRQoL-UK” scores according to time, group, and gender variables (*N* = 120).

Effect	Type III sum of squares	*df*	*F*	P^*^	* $\eta_{p}^{2}$ *
ADR					
Time^*^Group	1,951.426	6	59.653	**< 0.001**	0.61
Time^*^ Gender	2.880	2	0.264	0.768	0.00
Time^*^Group^*^Gender	34.695	6	1.061	0.387	0.02
OHRQoL-UK					
Effect					
Time^*^Group	17,680.337	4.63	174.334	**< 0.001**	0.82
Time^*^ Gender	67.627	1.54	2.000	0.149	0.01
Time^*^Group^*^Gender	154.158	4.63	1.520	0.190	0.03

ADR, Oral Assessment Guide; OHQoL-UK, Oral Health-Related Quality of Life Scale-United Kingdom; ^*^mix-design ANOVA; ηp^2^, Partial Eta Squared. Bold *p*-values indicate that the *p*-value is statistically significant.

**Figure 2:**
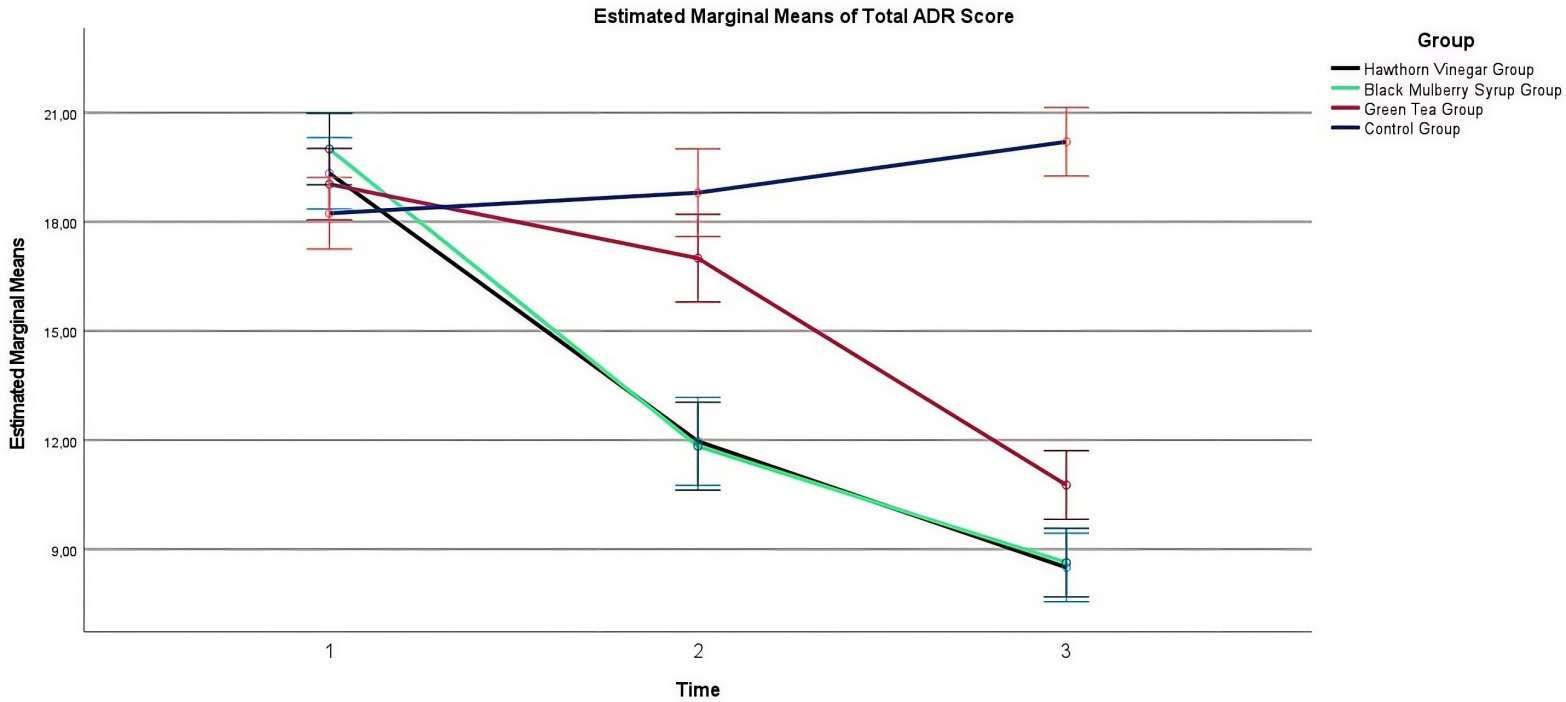
Interaction graph of Group*Time Factors on total ADR.

The mixed-design ANOVA analyses performed in Figure 2 revealed statistically significant differences between the experimental groups (hawthorn vinegar, black mulberry syrup, green tea) and the control group (*F* = 59.653; *p* < 0.001). When the Time^*^Group interaction was examined, it was determined that the hawthorn vinegar, black mulberry syrup, and green tea groups showed a significant decrease compared to the control group. However, while no difference was found between the hawthorn vinegar and black mulberry syrup groups (*p* > 0.05), these two natural products were found to be more effective than green tea in reducing the total ADR score (*p* < 0.05; Figure 2).

**Figure 3:**
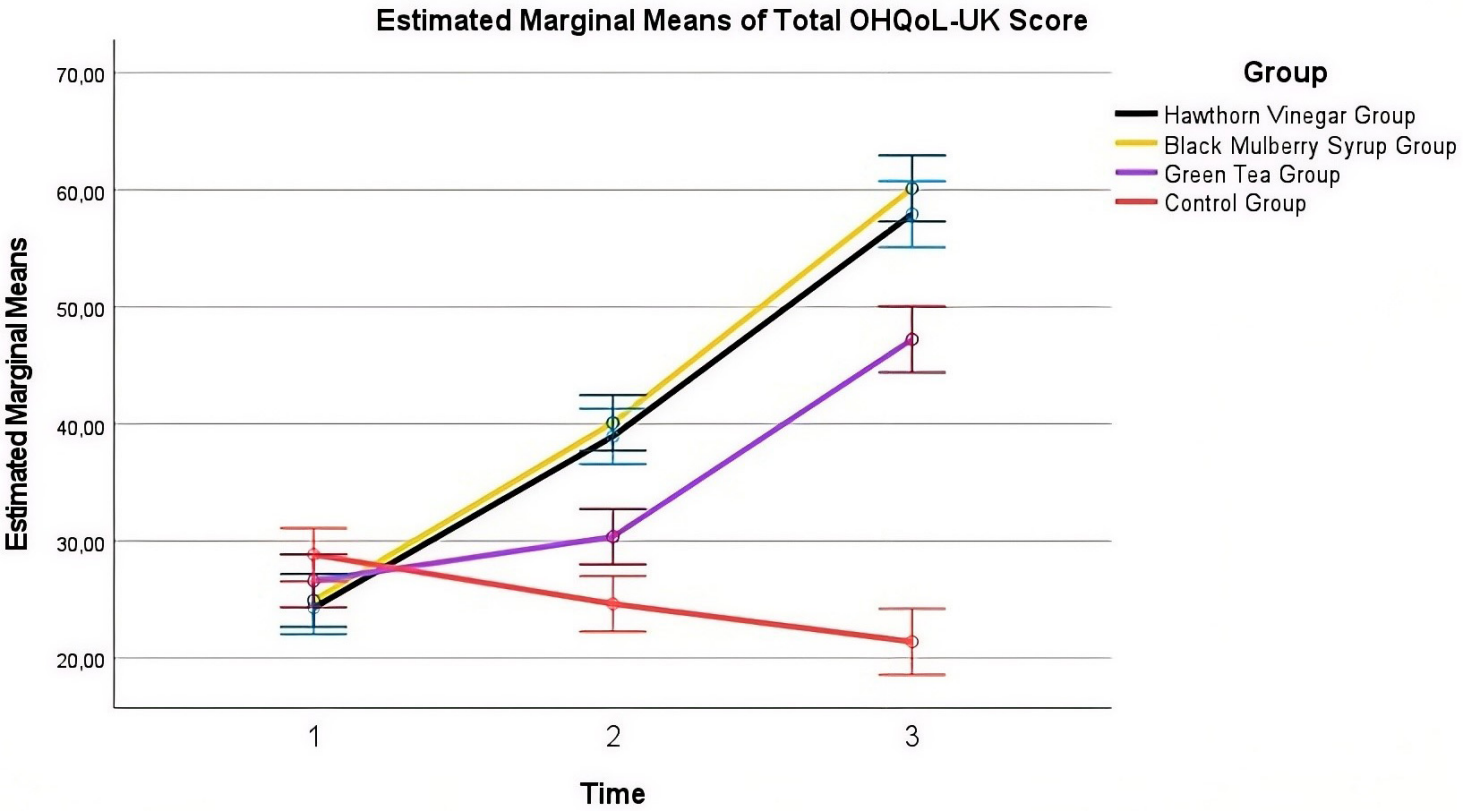
Interaction graph of Group*Time Factors on total OHQoL-UK.

The mixed-design ANOVA analyses performed in Figure 3 revealed statistically significant differences between the experimental groups (hawthorn vinegar, black mulberry syrup, green tea) and the control group (*F* = 174.334; *p* < 0.001). When the Time^*^Group interaction was examined, it was determined that the hawthorn vinegar, black mulberry syrup, and green tea groups increased significantly compared to the control group. However, while no difference was found between the hawthorn vinegar and black mulberry syrup groups (*p* > 0.05), these two natural products were found to be more effective than green tea in increasing the Total OHQoL-UK score (*p* < 0.05; Figure 3).

### 3.1 Safety and tolerability

Regarding the safety profile of the study, no allergic reactions, adverse events, or complications (such as oral irritation, severe pain, or persistent taste intolerance) were observed or reported by the research team or patients throughout the study period.

## 4 Discussion

This study is the first experimental research evaluating the effect of hawthorn vinegar, black mulberry syrup, and green tea on oral mucositis and oral health-related quality of life in individuals with Type 2 diabetes. No other study comparing these three interventions has been found in the literature. Therefore, the findings were discussed in relation to existing studies in the field of cancer and oral diseases. However, the high tolerance observed to hawthorn, black mulberry, and green tea in the study, as well as the absence of irritation or pain, can be attributed to the standardized dilution method used for the solutions. It is thought that the dilution method used in the study minimized the acidity of the vinegar or the bitterness of the tea while preserving their therapeutic properties.

The high tolerance observed in this study to hawthorn vinegar, black mulberry syrup, and green tea applications, along with the absence of irritation or pain, can be attributed to the standardized dilution method used for the solutions. It is thought that the dilution method minimizes the acidity of the vinegar and the bitterness of the tea while preserving their therapeutic properties. However, certain clinical and methodological precautions should be considered when evaluating these findings. Theoretically, the acidic nature of vinegar-based gargles has the potential to cause irritation to the mucous membranes, a burning sensation in lesion areas, or taste intolerance with repeated use; furthermore, the risk of allergy due to herbal ingredients should not be ignored. Findings on the effectiveness of these interventions in the current literature generally come from different populations, such as cancer or COPD patients, and the level of evidence in individuals with type 2 diabetes is still limited. Furthermore, the single-center nature of our study, per-protocol analysis, and deviations from the initial sample target may affect the generalizability of the conclusions that hawthorn vinegar and black mulberry syrup are more effective than green tea. Therefore, the observed positive effects should be viewed not as a “definite superiority,” but as potential supportive approaches in the diabetic population and should be supported by larger sample-size, highly blinded studies.

In this study, a significant decrease in the severity of oral mucositis and an increase in oral health-related quality of life were observed on days 7 and 14 in the groups treated with hawthorn vinegar. The level of effectiveness was found to be similar to black mulberry syrup and superior to green tea. These results are consistent with similar studies in the literature (8, 25–27). The study by Türkben Polat and Bahçecioglu Turan (26) with chemotherapy patients revealed the positive role of apple cider vinegar and black mulberry in preventing and improving oral mucositis. Similarly, randomized controlled trials on grape vinegar by Hasheminasab et al. (25) and Afrasiabifar et al. (8, 27) proved that vinegar-based mouthwashes both reduced chemotherapy-related oral mucositis and improved quality of life related to oral health. Afrasiabifar et al. (27) determined that the effectiveness of a mixture of grape vinegar and rose water is equivalent to that of traditional chlorhexidine gargle. These studies explain these positive clinical outcomes with the antibacterial properties of vinegar and its potential to accelerate wound healing. The increase in the “symptoms” subscale scores of the OHQoL-UK in our study also supports this mechanism.

In the reviewed literature, Demir Dogan et al. (28) reported that black mulberry molasses reduced the severity and frequency of mucositis in head and neck cancer patients undergoing radiotherapy. Similarly, Korkut et al. (10) found that black mulberry syrup provided more significant improvement in mucositis and dry mouth compared to sodium bicarbonate in individuals with COPD, while Karabey et al. (29) found the same in cancer patients. Harman et al. (30) observed that black mulberry syrup minimized mucositis formation in patients undergoing stem cell transplantation compared to other solutions. Raghunand Sindhe et al.'s (31) systematic review also confirms black mulberry's effect in reducing advanced mucositis and improving quality of life. Yüce Sari et al. (32) reported that grape and black mulberry molasses have similar efficacy, while Çubukçu and Çinar (33) reported that black mulberry syrup is more effective in preventing the development of mucositis in patients undergoing chemotherapy. Demir et al. (34) also found that black mulberry molasses alleviated mucositis symptoms, including pain, in patients undergoing radiotherapy. In this study, a significant decrease in oral mucositis and an increase in oral health-related quality of life were observed on days 7 and 14 in the black mulberry syrup group, and its efficacy level was found to be similar to that of hawthorn vinegar and superior to that of green tea. These findings are consistent with studies in the literature.

This study found that green tea reduced oral mucositis and improved quality of life, but these effects were more limited compared to hawthorn vinegar and black mulberry syrup. Similar to the findings of our study, the literature reports that green tea improves ADR scores in oral cancer patients (9) and is an alternative to chlorhexidine in the treatment of pericoronitis (35). Furthermore, it has been found to have an antibacterial effect on Streptococcus mutans (36), improve bad breath (37), and be effective in denture stomatitis (38). Meta-analysis studies suggest that green tea may be an alternative treatment for radiation-induced mucositis (39), while systematic reviews support its ability to reduce dry mouth in periodontitis (23). The literature reports that green tea is a natural and safe oral health agent (9). These positive effects are explained by the antioxidant and anti-inflammatory properties of green tea's polyphenolic compounds (40).

Oral mucositis causes a decrease in patients' quality of life due to increased pain, decreased performance status, and inability to eat (28). In this study, a negative correlation was found between oral mucositis and oral health-related quality of life in the interim and final test measurements. This finding from our study is consistent with all the studies reviewed and supports the information in the literature. Furthermore, the statistical significance observed in ADR scores is supported by high effect sizes ($\eta_{\text{p}}^{2}$) detected in critical sub-dimensions such as swallowing, voice, and pain, indicating high practical importance for diabetic patients. Beyond the numerical data, the decreases in ADR scores directly represent functional improvement; for example, improvements in “swallowing” and “voice” scores reflect a transition from a stage of speech difficulty or painful swallowing to a stage where the patient can comfortably perform daily living activities. This clinical improvement also parallels a significant increase in the “physical condition” dimension of the OHQoL-UK scale, which measures the impact on eating and oral functions. Evaluation of these results together with confidence intervals (95% CI) proves that the observed improvement is not coincidental and that the clinical effect of the intervention is within a consistent range, even with a certain margin of uncertainty. Therefore, high effect sizes and narrow confidence intervals confirm that interventions such as hawthorn vinegar and black mulberry syrup have the potential to provide not only statistical data but also a noticeable improvement in patients' quality of life in mucositis management.

## 5 Conclusion and recommendations

In conclusion, this study suggests that hawthorn vinegar and black mulberry syrup may have potential benefits as complementary approaches in reducing the severity of oral mucositis and enhancing oral health-related quality of life in individuals with Type 2 DM. The findings indicate a significant negative correlation between oral mucositis levels and quality of life scores. While these interventions may be considered as supportive options alongside standard oral care, the results should be interpreted with caution due to the study's single-center design and the use of per-protocol analysis. To validate these preliminary findings and establish their clinical efficacy, further multicenter clinical trials with larger sample sizes, longer follow-up periods, and more robust blinding designs are required before these approaches can be formally integrated into routine clinical protocols.

### 5.1 Limitations

When evaluating the findings of this study, several methodological limitations that may affect the internal validity and generalizability of the results should be considered. First, the single-center design of the study and the voluntary inclusion of participants may have created a selection bias by leading to the inclusion of a group already positively inclined toward complementary approaches. The inability to achieve complete double-blindness at the participant level due to the unique sensory properties (taste, smell, and color) of the agents in the intervention groups (hawthorn vinegar, black mulberry syrup, and green tea) poses a risk of performance bias, particularly in subjective quality of life scores. Furthermore, the reduction in sample size from 140 to 120 in the analysis phase due to losses during the follow-up process, and the fact that the analysis was conducted on a “per-protocol” basis instead of an “intention-to-treat” basis, may have led to a focus only on data from participants showing high compliance, thus causing a compliance bias. Despite the limited sample size and possible baseline imbalances in some secondary outcomes, the high effect sizes observed in primary measures such as ADR (0.61) and OHRQoL-UK (0.82) confirm that the statistical power of the study is preserved; however, larger sample and multicenter studies are needed before these interventions can be incorporated into routine clinical protocols.

Another limitation of this study is the inability to completely blind the participants due to the unique sensory profiles of the natural products utilized. Finally, although the baseline gender distribution was perfectly homogenous across all study groups (*p* = 0.680), data were not disaggregated by sex/gender for sub-group analyses due to sample size constraints (*n* = 30 per group) and the lack of established gender-specific dimorphism in the biological mechanisms of the natural products tested. Future multi-center studies with larger cohorts are recommended to conduct sex-stratified analyses in line with SAGER guidelines.

## Data Availability

The raw data supporting the conclusions of this article will be made available by the authors, without undue reservation.
